# The Prognostic Value of a Naples Score in Determining in-Hospital Mortality in Patients with Acute Ischemic Stroke Undergoing Endovascular Treatment

**DOI:** 10.3390/jcm13216434

**Published:** 2024-10-27

**Authors:** Onur Kadir Uysal, Derya Ozdogru, Abdullah Yildirim, Ilker Ozturk, Guluzar Tras, Zulfikar Arlier

**Affiliations:** 1 Department of Cardiology, Adana City Training & Research Hospital, University of Health Sciences, 01230 Adana, Turkey; dr.yildirimabdullah@gmail.com (A.Y.); guluzar.tras@gmail.com (G.T.); 2 Department of Neurology, Adana City Training & Research Hospital, University of Health Sciences, 01230 Adana, Turkey; deryaozdogru@hotmail.com (D.O.); ilkerozturk01@hotmail.com (I.O.); zarlier@gmail.com (Z.A.)

**Keywords:** acute ischemic stroke, Naples prognostic score, inflammatory markers, neutrophil–lymphocyte ratio, endovascular stroke treatment

## Abstract

**Background/Objectives**: The Naples prognostic score (NPS), reflecting inflammation and nutritional status, has prognostic value, especially in cancer. This study evaluated its ability to predict in-hospital mortality in acute ischemic stroke (AIS) patients undergoing endovascular treatment (EVT). **Methods**: We retrospectively studied 244 patients with AIS who were admitted between April 2020 and December 2023. Patients were included if they presented within 6 h of symptom onset with evidence of intracranial proximal arterial occlusion. The EVT was performed using aspiration catheters, stent retrievers, or both. The NPS was calculated based on the neutrophil–lymphocyte ratio, lymphocyte–monocyte ratio, and albumin and total cholesterol levels. **Results**: We found a significant association between higher NPS scores and in-hospital mortality. Patients with a high NPS (3 or 4) had a mortality rate of 41.6% compared to 21.0% in the low-NPS group (0, 1, or 2). The full model incorporating NPS showed superior predictive ability for in-hospital mortality compared with the baseline model (areas under the curve 0.881 vs. 0.808). A receiver-operating characteristic analysis at a cutoff of >2.5 for the NPS showed a sensitivity of 86.6% and specificity of 41.9%. This study demonstrated that incorporating the NPS into the predictive model improved the accuracy and calibration for predicting in-hospital mortality. A decision curve analysis showed the net benefit of using the full model incorporating NPS over the baseline model, emphasizing its potential clinical application in prognostication. **Conclusions**: NPS is a reliable predictor of in-hospital mortality in AIS patients undergoing EVT. Incorporating NPS into clinical practice could help to identify high-risk patients and improve outcomes through tailored interventions.

## 1. Introduction

Stroke is one of the leading causes of death and disability worldwide. Acute ischemic stroke (AIS) due to thrombosis and embolism accounts for 87% of all stroke patients [1]. Endovascular treatment (EVT) is highly effective for selected patients with AIS. Many parameters are used to predict early clinical outcomes after EVT. These include duration of symptoms, age, clinical signs of cortical deficit, National Institute of Health Stroke Scale (NIHSS), Alberta Stroke Program Early Computed Tomography (ASPECT) scores, collaterals, glucose levels, and blood pressure, which have been demonstrated in previous studies [2,3].

Inflammatory responses play a major role in the pathophysiological mechanisms of AIS [4,5]. In several studies, inflammatory parameters such as the neutrophil–lymphocyte ratio (NLR), platelet–lymphocyte ratio (PLR), lymphocyte–monocyte ratio (LMR), and C-reactive protein (CRP) levels were found to be associated with early neurological deterioration in patients with AIS after thrombolysis, as well as poor outcomes and long-term functional deficits in patients with AIS undergoing EVT [6,7].

The Naples prognostic score (NPS) is a newly developed scoring system that assesses inflammatory and nutritional status, incorporating NLR, LMR, albumin, and total cholesterol (TC) levels, and is widely used in cancer patients [8]. While there have been many studies on NPS in patients with cancer, there are limited data on the use of NPS in cardiovascular diseases, and we could not find studies on its use in AIS. Recent studies have shown that NPS has a strong predictive role in ST-elevation myocardial infarction and heart failure and is superior to other inflammation markers and grading scores [9,10,11,12].

Therefore, we aimed to comprehensively examine the relationship between NPS and in-hospital mortality in AIS patients who underwent EVT.

## 2. Materials and Methods

### 2.1. Study Participants and Endovascular Procedure

**Figure 1:**
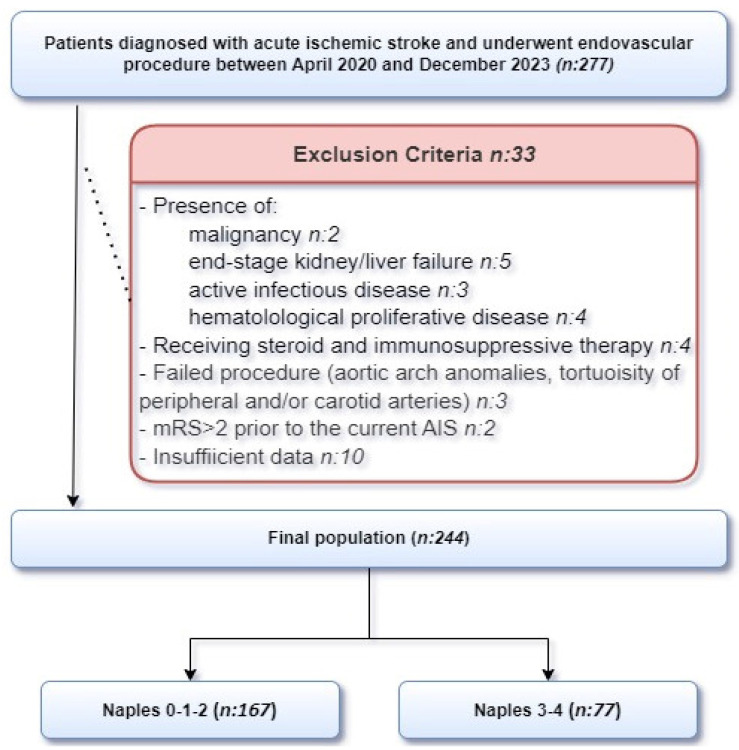
This flowchart illustrates the selection process of patients diagnosed with acute ischemic stroke who underwent endovascular procedures.

We conducted a retrospective, single-center study of patients with AIS admitted to the Neurovascular Unit at the University of Health Sciences Adana City Training and Research Hospital from April 2020 to December 2023. A total of 244 patients underwent either intravenous or intra-arterial thrombolysis with EVT or underwent EVT alone. Patients were selected for EVT according to the current American Heart Association/American Stroke Association guideline [13]. The EVT was performed on patients who were admitted to our hospital according to the following inclusion criteria: age ≥ 18 years; ASPECTS ≥ 6 and NIHSS ≥ 6; patients presenting to the emergency department within the first 6 h of symptom onset and with evidence of intracranial proximal arterial occlusion, specifically in the M1 and M2 segments of the middle cerebral artery; and internal carotid artery or basilar artery, confirmed by vascular imaging such as computed tomography and magnetic resonance imaging. The exclusion criteria were as follows: presence of malignancy (*n* = 2), end-stage kidney or liver failure (*n* = 5), active infectious disease (*n* = 3), hematological proliferative disease (*n* = 4), ongoing steroid or immunosuppressive therapy (*n* = 4), failed procedure due to aortic arch anomalies, tortuosity of the peripheral and/or carotid arteries (*n* = 3), modified Rankin Scale (mRS) score > 2 prior to the current AIS (*n* = 2), and insufficient data for analysis (*n* = 10). After applying the exclusion criteria, 244 patients were included in the final analysis (Figure 1).

All procedures were performed using a monoplane angiography machine (Siemens Healthineers; Forchheim, Germany). All the procedures achieved access by implanting an 8 Fr sheath into the femoral artery. The EVT consisted of mechanical thrombectomy using stent retrievers alone, thromboaspiration with catheters alone, or a combination of both, depending on the operational team choice as well as on the occlusion type and location. During the procedure, a heparinized saline solution was continuously perfused through the distal catheter. Following the procedure, the Thrombolysis in Cerebral Infarction (TICI) scale was used to evaluate its success. A TICI score of IIb or III was considered a successful recanalization result. The mRS was used to indicate the degree of disability or dependence on daily activities following the incident. In-hospital mortality was defined as all-cause death during hospitalization. Patient records and the National Health Registry were used to retrospectively gather each patient’s medical history and comorbidities. Hypertension was classified as having a systolic and diastolic blood pressure of ≥140 and 90 mmHg, respectively, or the use of oral antihypertensive medication. A history of coronary artery disease was defined as the presence of at least one of the following conditions: acute coronary syndrome, stent or balloon angioplasty, or coronary artery bypass grafting.

### 2.2. Laboratory Analysis and Naples Prognostic Score Calculation

The calculation of the inflammatory indices was based on venous samples obtained at the time of admission. The serum levels of neutrophils, lymphocytes, platelets, hemoglobin, CRP, and albumin, as well as lipid profiles and liver and kidney function parameters, were assessed using a Beckman UniCel DxC 800 Synchron autoanalyzer (Beckman Coulter Inc., CA, USA) and appropriate tubes. The NPS was calculated using four parameters, including NLR, LMR, albumin, and TC concentrations, as defined by Galizia et al. [8]. A level of NLR greater than 2.96, or LMR less than or equal to 4.44, TC less than or equal to 180 mg/dL, or albumin less than 4.0 mg/dL results in 1 point on the scale for each value within these parameters, and anything outside of these values does not constitute a point. The NPS was scored from 0 to 4. Those with an NPS of 0, 1, or 2 were classified as the low-NPS group, and those with an NPS of 3 or 4 were classified as the high-NPS group. The demographic, clinical, and procedural findings were compared between the high- and low-NPS groups.

### 2.3. Statistical Preparation

Statistical analyses were conducted using the R statistical software (version 4.3.2, Vienna, Austria). The normality of variables was assessed using a Kolmogorov–Smirnov test, supported by visual inspection of histograms and probability plots. Continuous variables are presented as mean ± standard deviation for normally distributed data and as median (interquartile range (IQR25-75)] for non-normally distributed data. Categorical data were expressed as numbers (percentages, %). Pairwise comparisons of categorical variables were performed using a Fisher’s exact test or χ^2^ test, while an independent Student’s *t*-test and Mann–Whitney U tests compared continuous variables between groups.

**Figure 2:**
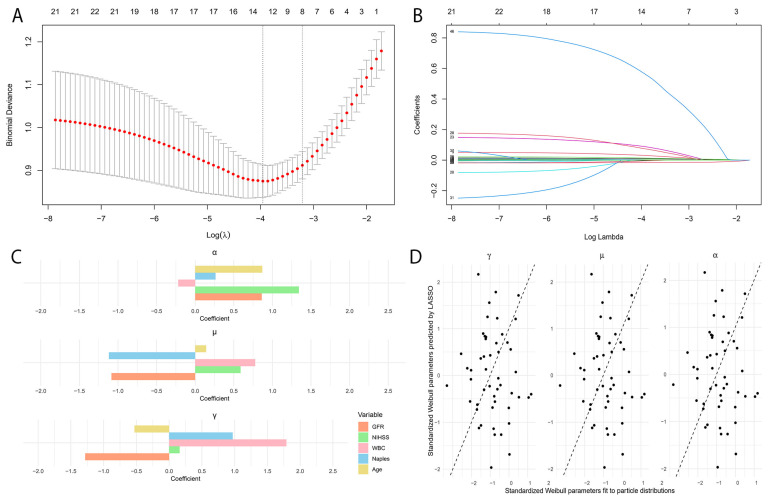
The least absolute shrinkage and section operator (LASSO)-penalized selection method analyses for the variable selection of the models. (**A**) The LASSO coefficient profiles were examined for various features, and the selection criterion for lambda was determined based on specific criteria, highlighting the regularization path of the LASSO algorithm in the context of our analysis. (**B**) The distribution of the minimum mean-squared error alongside the corresponding penalization lambda value in the LASSO-penalized model. (**C**) Bar charts representing standardized coefficient estimates for key variables across different model parameters, illustrating the impact and significance of each predictor within the model framework. (**D**) Scatter plots comparing standardized Weibull parameters predicted by LASSO with observed data distributions, used to assess the accuracy and predictive performance of the LASSO model.

A least absolute shrinkage and section operator (LASSO)-penalized selection method was applied to variables identified as significant in the logistic regression analysis, utilizing an optimal lambda value for variable reduction to prevent overfitting. Figure 2A shows the binomial deviance across various λ values, identifying the optimal λ at the point of minimal deviance. Figure 2B illustrates the regression coefficients against the log of λ, demonstrating that variable selection as certain coefficients approach zero with increasing λ. Figure 2C shows the regression coefficients for the selected variables, highlighting their relative importance in the model. Finally, Figure 2D compares the standardized Weibull parameters predicted by LASSO with those fitted to particle distributions to assess the accuracy of our model’s predictions. The selected variables, based on the minimum lambda value, included the NIHSS, white blood cell count, age, glomerular filtration rate, urea, CRP, glucose, and NPS.

The discriminative capability of the baseline model (LASSO parameters except NPS) was compared to that of the full model (NPS plus baseline model) through pairwise comparisons of receiver-operating characteristic (ROC) curves using a DeLong method for predicting in-hospital mortality. Odds ratio (OR), adjusted odds ratio (aOR), and 95% confidence interval (CI) were obtained for all regression analyses. The model’s performance was assessed using various metrics, including a Brier score (lower values indicating better calibration, with values closer to 0 reflecting more accurate probability predictions), Akaike information criteria (lower values indicating better fit), adjusted R^2^ (higher values indicating better fit), and areas under the curve (AUCs) (<0.50, no discrimination; >0.75, good discrimination). ROC curves and AUCs were examined to determine the discriminative accuracy and performance of NPS. The optimal cutoff value for the NPS was determined using the maximum Youden index. Decision curve analysis illustrated the net benefit of using the full model over all treatment and non-treatment strategies, as well as the baseline model, for in-hospital mortality. Multicollinearity was assessed using the variance inflation factor with a threshold of >3 indicating significant multicollinearity. The goodness-of-fit of the logistic regressions was evaluated using a Hosmer–Lemeshow test. All statistical analyses used two-sided tests with a significance level (alpha) of 0.05.

## 3. Results

### 3.1. Baseline Characreristics

**Figure 3:**
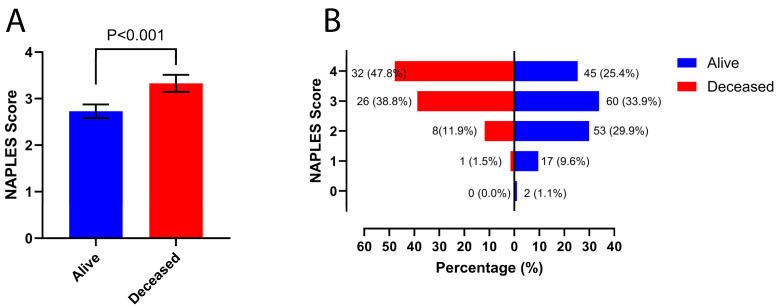
Naples scores were compared between alive and deceased patients. (**A**) The bar graph shows the mean (%) Naples scores for both groups, with the bars representing the standard error of the mean. (**B**) The horizontal bar chart illustrates the distribution of Naples scores for both groups, showing a higher prevalence of elevated scores among deceased patients compared to alive patients.

The baseline characteristics of the study population are presented in Table 1. The mean age of the 244 patients included in the study was 65.9 ± 12.2 years, and 56.6% were female. Among the groups analyzed by NPS, there were no significant differences in baseline characteristics, including age, sex, BMI, and the prevalence of conditions such as hypertension and prior atrial fibrillation. Regarding laboratory parameters, urea (P = 0.001) and CRP (P < 0.001) levels were also higher in the high-NPS group. LDL-C (P < 0.001), HDL-C (P < 0.001), lymphocyte (P < 0.001), and hemoglobin (P = 0.004) levels were higher in the low-NPS group. There was no significant difference between the two groups in terms of periprocedural clinical parameters, including the time from symptom onset to presentation at the emergency department, door-to-femoral puncture time, and use of tPA. The occlusion area and procedural techniques were similar between the groups. In the high-NPS group, TICI 0 and ≤IIa rates were slightly higher, whereas TICI IIb and III rates were slightly lower; however, these differences were not statistically significant. In-hospital mortality occurred in 67 (27.5%) patients. Mortality rates were more frequent in the group with a high NPS (21.0 vs. 41.6%, P < 0.001). When participants were divided into groups according to their mortality status, the mean NPS was higher in the group with a higher mortality rate (P < 0.001) (Figure 3).

### 3.2. Model Performance and Naples Score

**Figure 4:**
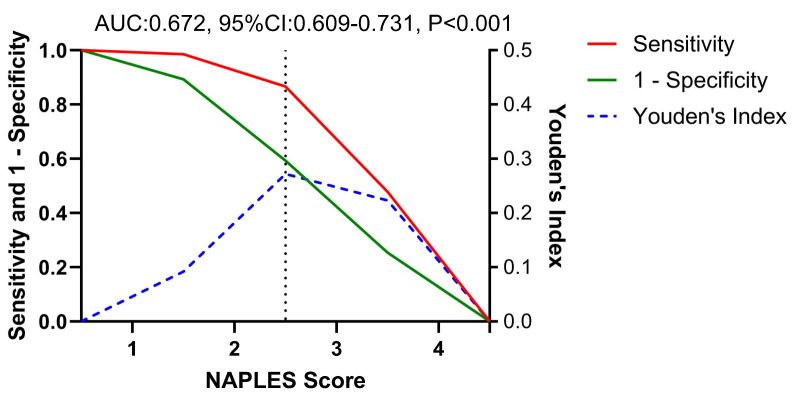
ROC curve analysis for Naples score showing the relationship between sensitivity (red line) and 1-specificity (green line). Youden’s Index (blue dashed line) is also plotted to indicate the optimal cut-off point.

The ROC curve analysis was performed with in-hospital mortality as the state variable and NPS as the test variable. The ROC curve analysis at a cutoff of >2.5 (according to the maximal Youden’s Index) for NPS revealed a sensitivity of 86.6% and specificity of 41.9% in detecting in-hospital mortality (AUC = 0.672, standard error = 0.191, 95% CI: 0.609–0.731, P < 0.001) (Figure 4).

**Figure 5:**
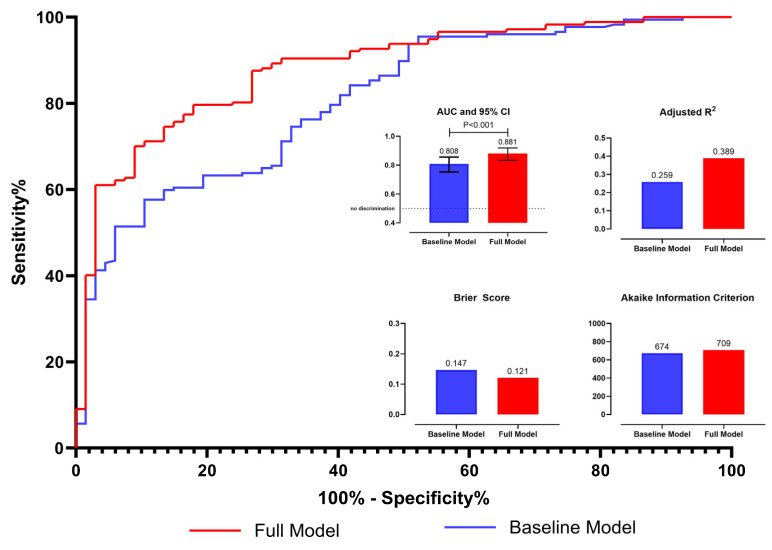
The ROC curves for the full model and the baseline model are shown. Insets display performance metrics: AUC, Adjusted R^2^, Brier Score, and Akaike Information Criterion (AIC), illustrating the comparative performance of the two models.

**Figure 6:**
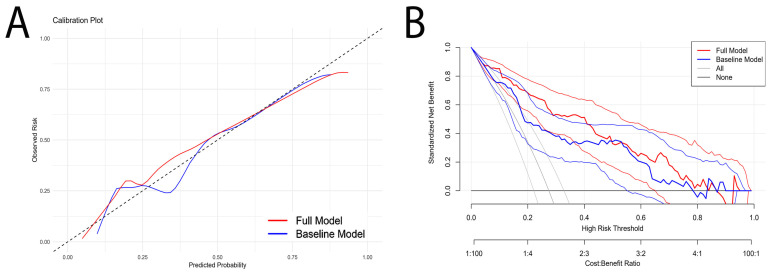
(**A**) Calibration plot comparing the observed risk against the predicted probability for the full model and the baseline model. (**B**) Decision curve analysis displaying the standardized net benefit of the full model and the baseline model across a range of high-risk thresholds. The curves for treating all patients (gray line) and treating none (black line) are also included for comparison.

The ROC curve analysis demonstrated that the full model incorporating NPS exhibited superior discriminative ability compared to the baseline model (AUC 0.881 vs. 0.808, P = < 0.001; Akaike information criterion 709 vs. 674; adjusted R^2^ index 0.389 vs. 0.259; Brier score 0.121 vs. 0.147) (Figure 5). Similarly, the incorporation of NPS into the model significantly improved its calibration (Figure 6A). Additionally, the decision curve analysis depicted the net benefit of using the full model, incorporating NPS, over the base model for in-hospital mortality (Figure 6B).

When the individual parameters of NPS were examined in more detail by regression analysis, LMR (a-OR = 2.767, 95% CI: 0.771–9.29, P = 0.119), NLR (a-OR = 4.066, 95% CI: 1.460–11.327, P = 0.007), TC (a-OR = 2.852, 95% CI: 1.289–6.311, P = 0.010), and serum albumin (a-OR = 1.016, 95% CI: 0.439–2.352, P = 0.971) were independently associated with in-hospital mortality in patients with AIS undergoing EVT (Table 2).

### 3.3. Subgroup Analyses

**Figure 7:**
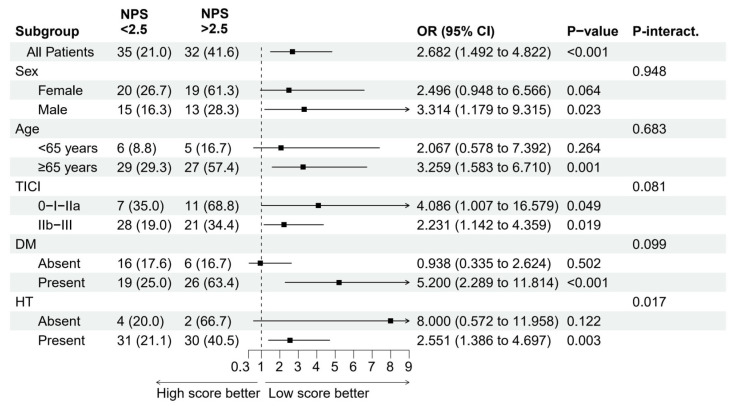
Forest plot and subgroup analysis of in-hospital mortality based on NPS (<2.5 vs. >2.5). The p-interaction value represents the probability of interaction between NPS and other categoric variables.

In the subgroup analyses (Figure 7), there was a significant difference between the low- (≤2.5) and high-NPS (>2.5) groups in determining in-hospital mortality in subgroups of participants with males (OR = 3.314, 95% CI 1.179–9.315, P = 0.023), patients aged ≥65 years (OR = 3.259, 95% CI 1.583–6.710, P = 0.001), patients with TICI 0-I-IIa (OR = 4.086, 95% CI 1.007–16.579, P = 0.049), patients with diabetes mellitus (OR = 5.200, 95% CI 2.289–11.814, P < 0.001), and patients with hypertension (OR = 2.551, 95% CI 1.386–4.697, P = 0.003). Conversely, no significant differences were found for females (OR = 2.496, 95% CI 0.948–6.566, P = 0.064), patients aged <65 years (OR = 2.067, 95% CI 0.578–7.392, P = 0.264), and patients without diabetes mellitus or hypertension.

## 4. Discussion

This study is the first to evaluate the relationship between the NPS and in-hospital mortality in patients with AIS after EVT. Patients with a higher NPS had higher mortality rates than those with a lower NPS. The analysis demonstrated that NPS had a strong ability to identify patients at a risk of in-hospital mortality. The full model, which included NPS, showed better discrimination and calibration than those of the baseline model. Furthermore, decision curve analysis highlighted the benefit of using the full model over the baseline model in predicting in-hospital mortality. These results suggest that NPS may serve as an important independent predictor of prognosis in patients with AIS.

Studies have demonstrated that inflammation plays a crucial role in the pathogenesis of many diseases [14]. Additionally, the inflammatory process is highly significant in the development of atherosclerosis, which is a crucial cause of all cardiovascular diseases. Moreover, in AIS, it has been stated that the inflammatory response begins within 24 h in the ischemic area and that inflammation contributes to the exacerbation of ischemic damage [15]. Inflammation induced by reduced cerebral blood flow has the potential to enhance brain tissue damage through the activation of intravascular leukocytes and pro-inflammatory mediators released from the endothelium. Leukocytes in ischemic brain tissue adhere to endothelial cells via adhesion molecules, followed by chemokine-activated pro-inflammatory cytokines, leading to an inflammatory cascade response [16,17]. Neutrophils migrate to cross the blood–brain barrier and have been shown to invade within 1 h, causing brain tissue damage, which peaks 2–3 days after a stroke and remains present for approximately 14 days [18]. Neutrophils and monocytes can activate matrix metalloproteinase-9 and induce free oxygen radicals, which can cause clinical deterioration, hemorrhagic transformation, and brain edema in AIS [19]. In contrast to neutrophils, it has been reported that some lymphocyte subtypes have significant cerebroprotective effects in response to ischemic damage after AIS via the release of anti-inflammatory cytokines and stimulation of neuroangiogenesis [20]. Thus, they play a role in reducing infarct size and improving long-term neurological function [21].

Several studies have suggested that composite inflammatory indicators, including NLR, PLR, and LMR, might be associated with clinical outcomes in AIS or transient ischemic attack, hemorrhagic transformation after stroke, and symptomatic internal carotid artery stenosis [22,23,24]. In addition, NLR and LMR were found to predict the 3-month functional outcome in patients with AIS undergoing EVT [22]. Recently, Zhu et al. demonstrated that NLR is a predictor of poor short-term prognosis in patients with AIS [25]. In our study, we found that NLR and LMR, which are components of NPS, were significantly associated with in-hospital mortality. In the multivariable logistic regression analysis, we concluded that the predictive power of NPS for mortality in this patient group was mainly due to NLR. As mentioned above, the significant increase in NLR, a good marker of inflammation, in the group of patients with high mortality was consistent with the pathophysiological processes we discussed and the current studies.

Albumin and TC are used as biomarkers of nutritional status. Nutritional risk was assessed using the Geriatric Nutritional Risk Index (GNRI), Prognostic Nutritional Index (PNI), and Controlling Nutritional Status (CONUT) models based on laboratory test data (i.e., serum albumin, lymphocyte count, and TC). In AIS patients, malnutrition is a predictor of poor clinical outcomes in both short- and long-term periods [26]. Moreover, nutritional status is associated with comorbidities such as inflammation, infection, disease progression, and complications during hospitalization [27]. Ha et al. reported that a high TC level was independently associated with a better clinical outcome (mRS 0–2) at the 3-month follow-up in AIS patients after EVT, particularly in patients with cardioembolism and complete recanalization [28]. In addition, Koton et al. concluded that patients with TC less than 115 mg/dl have a high risk of stroke severity and worse functional outcomes, regardless of statin use before stroke [29]. A possible hypothesis for the beneficial effect of TC is that when the blood–brain barrier is damaged by ischemia, TC may improve the repair and remyelination of the penumbral tissue [30]. TC may also have a buffering effect on free radicals released during ischemic damage, thereby limiting the extent of necrosis [31]. In our study, TC levels below 180 mg/dL, which is a component of NPS, were strong predictors of in-hospital mortality.

As a combined-score model, NPS may better reflect the immune and nutritional status of patients than its constituent parameters. It has been shown that markers of inflammation (NLR, PLR, and LMR) and nutritional status (GNRI, PNI, CONUT) are also individually significant predictors of in-hospital mortality and worse clinical outcomes in patients with AIS [7,32]. To the best of our knowledge, no published studies have investigated the role of NPS in stroke patients. In recent studies, NPS was found to be associated with left ventricular ejection fraction and SYNTAX score, which are important predictors of mortality in patients with ST-segment elevation myocardial infarction [12,33,34]. In a recent study evaluating the predictive power of the Systemic Immune Inflammation Index and NPS in detecting the severity of coronary artery disease, adding the NPS to the model did not perform better than the baseline statistical model [35]. In our study, the use of the NPS, in addition to strong and known specific prognostic parameters such as age, CRP, NIHSS, and white blood cell count, increased the power of the baseline model. Moreover, in the statistical modeling performed in our study, which compared the discriminative capability of the baseline model and the full model for predicting in-hospital mortality, the full model predicted in-hospital mortality better than baseline. We believe that incorporating the NPS into models alongside known risk factors will enhance prognostic accuracy in stroke patients. In addition, the findings of the decision curve analysis showed the net benefit of using the full model for in-hospital mortality in our patient group. These findings may have implications for the early prediction of poor outcomes and mortality as well as the improvement of clinical outcomes in patients with AIS who have undergone EVT.

Our study has several limitations. The most important limitations of this study include that it was performed retrospectively and that it comprised a relatively small sample size. Therefore, the predictive value of NPS should be further investigated in prospective studies with larger sample sizes.

Also, we did not have long-term follow-up data, including the mRS and mortality rates at 6 months. Had we had access to these data, we would have had more information about the clinical predictive value of NPS.

Moreover, we excluded diseases such as malignancy, hematological proliferative disease, ongoing steroid or immunosuppressive therapy, and active infectious diseases, which have been shown in the literature to affect inflammatory or nutritional (NLR, LMR, albumin, TC) parameters that compose the NPS. So, we have no data that the NPS can be used in these patient groups.

## 5. Conclusions

This study revealed that NPS is associated with an increased risk of mortality in patients with AIS who undergo EVT. An elevated NPS can predict groups at higher risk of poor clinical outcomes and in-hospital mortality.

## Figures and Tables

**Table 1:** Demographic and baseline data by groups of NaplesNaples score.

	All Patients (*n* = 244)	Naples 0-1-2 (*n* = 167)	Naples 3-4 (*n* = 77)	P-Value *
Age, Years	65.9 ± 12.2	65.87 ± 12.47	66.17 ± 11.78	0.859
Gender, female, *n* (%)	106 (56.6)	75 (44.9)	31 (40.3)	0.579
BMI, kg/m^2^	26.74 ± 4.21	26.43 ± 3.81	27.43 ± 4.95	0.085
Coronary artery disease, *n* (%)	85 (34.8)	62 (37.1)	23 (29.9)	0.312
Prosthetic heart valve, *n* (%)	16 (6.0)	10 (6.0)	6 (7.8)	0.587
Prior atrial fibrillation, *n* (%)	75 (30.7)	55 (32.9)	20 (26.0)	0.299
Diabetes mellitus, *n* (%)	117 (48.0)	76 (45.5)	41 (53.2)	0.273
Hypertension, *n* (%)	167 (68.4)	92 (55.1)	38 (49.4)	0.404
Previous stroke or TIA, *n* (%)	79 (32.4)	52 (31.1)	27 (35.1)	0.542
Current smoker, *n* (%)	15 (6.1)	11 (6.6)	4 (5.2)	0.781
Congestive heart disease, *n* (%)	61 (25.0)	42 (25.1)	19 (24.7)	0.937
Laboratory Parameters				
Glucose, mg/dL	137.5 ± 54.6	134.0 ± 53.5	145.2 ±56.7	0.138
Urea, mg/dL	42.8 ± 23.8	39.4 ± 13.7	50.4 ± 36.3	0.001
Creatinine, mg/dL	0.90 ± 0.43	0.87 ± 0.26	0.98 ± 0.67	0.061
GFR ^1^, mL/min/1.73 m^2^	82.9 ± 25.1	84.0 ± 23.6	80.5 ± 28.2	0.312
CRP, mg/L	5.8 (2.7–16.0)	4.7 (2.4–13.0)	7.8 (5.7–37.2)	<0.001
LDL-C, mg/dL	112.2 ± 34.6	118.6 ± 37.1	98.5 ± 23.2	<0.001
HDL-C, mg/dL	44.9 ± 12.6	47.1 ± 13.0	40.3 ± 10.2	<0.001
Triglyceride, mg/dL	96.7 ± 53.2	99.6 ± 57.2	90.7 ± 43.1	0.228
WBC, 10^3^/µL	10.10 ± 3.57	9.87 ± 3.72	10.64 ± 3.29	0.125
Lymphocyte, 10^3^/µL	1.66 ± 0.98	1.50 (1.00–2.50)	1.20 (0.80–1.40)	<0.001
Hemoglobin, g/dL	12.6 ± 1.9	12.9 ± 1.7	12.2 ± 2.2	0.004
Hematocrit, %	38.3 ± 5.2	38.9 ± 4.9	36.9 ± 5.9	0.007
Platelet count, 10^3^/µL	242 ± 84	235 ± 71	255 ± 105	0.087
Components of Naples Score				
Lymphocyte/monocyte ratio	2.3 (1.5–3.8)	2.8 (1.6–4.2)	1.8 (1.3–2.4)	<0.001
Neutrophil/lymphocyte ratio	5.2 (2.8–8.8)	4.07 (2.1–7.5)	6.8 (4.5–11.2)	<0.001
Albumin, mg/dL	3.75 ± 0.45	3.85 ± 0.46	3.52 ± 0.33	<0.001
Total cholesterol, mg/dL	169.0 ± 47.7	181.3 ± 51.6	142.3 ± 20.4	<0.001
Clinical Parameters				
*Cerebral edema*, *n* (%)	51 (20.9)	30 (18.0)	21 (27.3)	0.127
ASPECT	9.0 ± 0.9	9.0 ± 1.0	9.1 ± 0.9	0.871
Door to imaging time, min	16 ± 8	16 ± 8	16 ± 10	0.582
Time to recanalization, min	56 ± 19	55 ± 19	57 ± 18	0.563
Onset of symptom, min	120 (63–182)	117 (62–180)	125 (68–188)	0.287
Hemorrhagic transformation, *n* (%)	75 (30.7)	48 (28.7)	27 (35.1)	0.371
Intraarterial tPA, *n* (%)	16 (6.6)	11 (6.6)	5 (6.5)	0.965
Intravenous tPA, *n* (%)	5 (2.0)	5 (3.0)	0 (0.0)	0.329
Length of hospital stay, days	7 (5–10)	6 (5–9)	8 (6–11)	0.055
In-hospital mortality, *n* (%)	67 (27.5)	35 (21.0)	32 (41.6)	0.001
mRS at discharge	4 (1–6)	3 (1–4)	4 (2–6)	0.001
Stent retriever, *n* (%)	117 (48.0)	80 (47.9)	37 (48.1)	0.940
Thromboaspiration, *n* (%)	219 (89.8)	150 (89.8)	69 (89.6)	0.960
Baseline NIHSS	18.1 ± 3.6	17.9 ± 3.8	18.5 ± 3.1	0.229
Localization of occlusion, *n* (%)				0.295
ACA	1 (0.4)	1 (0.6)	0 (0.0)	
Basilar	5 (2.0)	3 (1.8)	2 (2.6)	
CCA	1 (0.4)	1 (0.6)	0 (0.0)	
ICA	61 (25.0)	37 (22.2)	24 (31.2)	
MCA proximal	117 (48.0)	84 (50.3)	33 (42.9)	
MCA distal	16 (6.6)	14 (8.4)	2 (2.6)	
PCA	3 (1.2)	3 (1.8)	0 (0.0)	
Tandem occlusion	38 (15.6)	23 (13.8)	15 (19.5)	
Vertebral artery	2 (0.8)	1 (0.6)	1 (1.3)	
TICI ^2^, *n* (%)				0.287
0	23 (9.4)	12 (7.2)	11 (14.3)	
I-IIa	13 (5.3)	8 (4.8)	5 (6.5)	
IIb	62 (25.4)	45 (26.9)	17 (22.1)	
III	146 (59.8)	102 (61.1)	44 (57.1)	

Data are shown in *n* (%), median (interquartile range; 25th–75th percentiles), and mean ± standard deviation. * A P-value of <0.05 was considered statistically significant. ^1^ Calculated according to the Chronic Kidney Disease Epidemiology Collaboration (CKD-EPI) equation. ^2^ Thrombolysis in cerebral infarction. grade 0: no perfusion; grade I: penetration with minimal perfusion; grade IIa: only partial filling (less than two-thirds) of the entire vascular territory is visualized; grade IIb: complete filling of all of the expected vascular territory is visualized but the filling is slower than normal; grade III: complete perfusion. Abbreviations; BMI: body mass index, ACA: Anterior cerebral artery, ASPECT: the Alberta Stroke Program Early Computed Tomography, GFR: glomerular filtration rate, HDL-C: high-density lipoprotein cholesterol, ICA: internal carotid artery, LDL-C: low-density lipoprotein cholesterol, MCA: median cerebral artery, mRS: Modified Rankin Scale, NIHSS: National Institutes of Health Stroke Scale, PCA: posterior cerebral artery, TIA: transient ischemic attack, TICI: Thrombolysis in cerebral infarction, tPA: tissue plasminogen activator, WBC: white blood cell.

**Table 2:** Multivariable logistic regression analysis of components of the Naples score that may have an independent association with in-hospital mortality.

Components of Naples Score	Adjusted Odds Ratio ^1^ (95% CI)	P-Value *
Lymphocyte/monocyte ratio-decrease from 4.44 to 0.53	2.767 (0.771–9.929)	0.119
Neutrophil/lymphocyte ratio-increase from 2.96 to 29.40	4.066 (1.460–11.327)	0.007
Total cholesterol-decrease from 180 mg/dL to 86 mg/dL	2.852 (1.289–6.311)	0.010
Serum albumin-decrease 4.0 mg/dL to 2.1 mg/dL	1.016 (0.439–2.352)	0.971

* A P-value of <0.05 was considered statistically significant. ^1^ Statistical performance; Hosmer-Lemeshow test P-value = 0.144, Nagelkerke R square = 0.410, −2 Log likelihood = 205.4, Model Chi-square = 81.415.

## Data Availability

Data are available on request due to privacy and ethical restrictions.
